# Surpassing light-induced cell damage *in vitro* with novel cell culture media

**DOI:** 10.1038/s41598-017-00829-x

**Published:** 2017-04-12

**Authors:** John H. Stockley, Kimberley Evans, Moritz Matthey, Katrin Volbracht, Sylvia Agathou, Jana Mukanowa, Juan Burrone, Ragnhildur T. Káradóttir

**Affiliations:** 1 Wellcome Trust-Medical Research Council Cambridge Stem Cell Institute & Department of Veterinary Medicine, University of Cambridge, Cambridge, CB2 1QR United Kingdom; 2 MRC Centre for Developmental Neurobiology, King’s College London, London, SE1 1UL United Kingdom; 3 Wellcome Trust-MRC Cambridge Stem Cell InstituteDepartment of Paediatrics, Cambridge Biomedical Campus, The Clifford Allbutt Building, Level 6, Hills Road, Cambridge, CB2 0AH UK

## Abstract

Light is extensively used to study cells in real time (live cell imaging), separate cells using fluorescence activated cell sorting (FACS) and control cellular functions with light sensitive proteins (Optogenetics). However, photo-sensitive molecules inside cells and in standard cell culture media generate toxic by-products that interfere with cellular functions and cell viability when exposed to light. Here we show that primary cells from the rat central nervous system respond differently to photo-toxicity, in that astrocytes and microglia undergo morphological changes, while in developing neurons and oligodendrocyte progenitor cells (OPCs) it induces cellular death. To prevent photo-toxicity and to allow for long-term photo-stimulation without causing cellular damage, we formulated new photo-inert media called MEMO and NEUMO, and an antioxidant rich and serum free supplement called SOS. These new media reduced the detrimental effects caused by light and allowed cells to endure up to twenty times more light exposure without adverse effects, thus bypassing the optical constraints previously limiting experiments.

## Introduction

For 40 years, ambient light has been known to be toxic for cells *in vitro* ^1^ and yet recent advances in new methodologies utilizing hazardous levels of light for non-invasive control of cells have rapidly evolved, such as optogenetics^2^, super-resolution imaging^3, 4^, ion and voltage sensitive imaging^5^, live cell imaging^4, 6–9^ and light triggered drug delivery^10^. The photo-toxicity associated with these methods is often underestimated, but can interfere with data accuracy and compromise experimental setups preventing their universal application^11, 12^. All visible wavelengths of light can be toxic to cells *in vitro* ^7^, dependent upon the dose and the wavelength, with the more energetic shorter wavelengths of light below 500 nm being particularly detrimental^9^. Optogenetics is a branch of synthetic biology involving the genetic introduction of light sensitive actuators to control neurotransmission^13^, subcellular signalling cascades^14, 15^, and gene regulation with temporal and spatial resolution^16–18^. Common optogenetic actuators such as channelrhodopsin-2 (ChR2)^13^, melanopsin (OPN4)^19^, cryptochrome-2 (Cry2)^15^, and light-oxygen sensitive proteins (LOV)^15^ rely on blue light for their photo-activation. The use of green fluorescent protein (GFP)^20^ as a fluorescent tag in live cell imaging also requires blue light excitation^20^, demonstrating broad applications of this particular wavelength of visible light. Thus, to fully utilize the advantages of these novel methods, we have focused on finding efficient solutions to blue light (470 nm) induced toxicity; by utilizing neural cells due to their sensitivity to light and the exponential increase in the usage of light stimulations in neuroscience.

## Results

### The effects of light on primary CNS cultures

**Figure 1:**
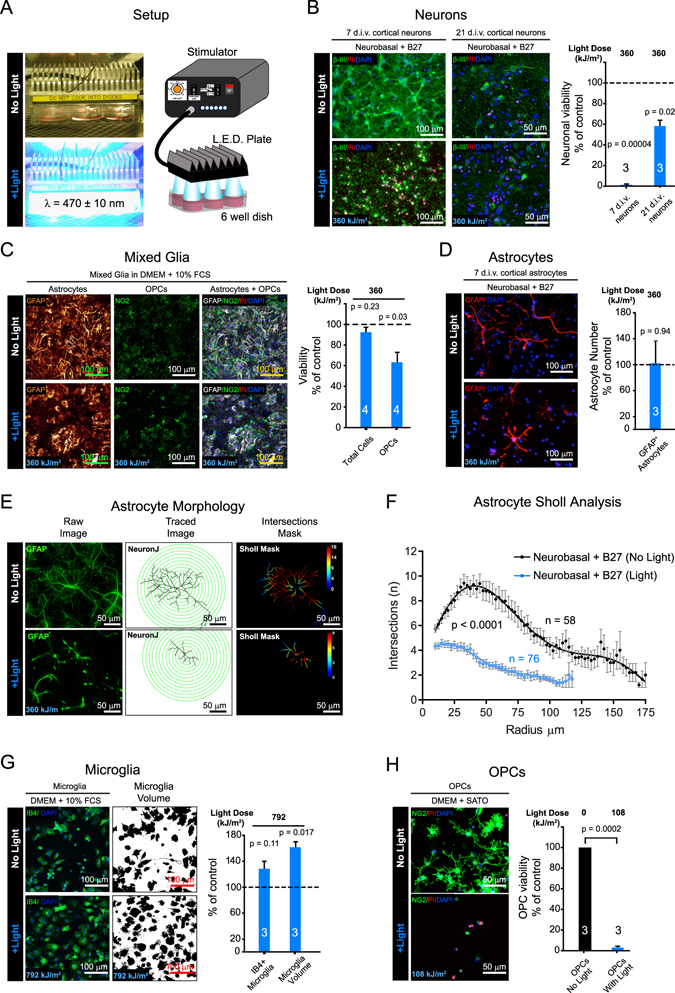
Light induces cytotoxicity *in vitro*. (**a**) Blue light (470 ± 10 nm) LED setup with control unit regulating delivery of power, pulse duration and frequency of light to cells in 6 well dish. (**b**) Viabilities of cortical neurons (β–III-tubulin^+^ (β–III)) of 7 or 21 days *in vitro* (d.i.v.) using propidium iodide (PI) exclusion assay after ± light at indicated light dose (units of kJ/m^2^). (**c**) Mixed glia (astrocytes (GFAP^+^) and OPCs (NG2^+^)) viabilities determined using PI exclusion assay after ± light treatment. (**d**) Representative images and quantification of GFAP^+^ astrocyte numbers in 7 d.i.v. cortical neuron enriched cultures after ± light at indicated dose. (**e**) Example of astrocyte morphological tracings after ± light treatment using GFAP staining and NeuronJ tracing and concentric radii of 10 µm steps overlaid in green to aid the reader. Sholl intersection masks with heat map of intersection number inset, generated using Sholl Analysis software from NeuronJ tracings. (**f**) Non-linear fitted plots of data from Sholl Analysis of astrocytes kept in the dark (black line: 58 cells analyzed) or exposed to light (blue line: 76 cells analyzed) and *p* value calculated from two tailed unpaired t-test of mean intersection number. (**g**) Microglial numbers (IB4^+^ cells) and volumes from binarised image masks of IB4^+^ cells using ImageJ and expressed as percentage area after ± light. (**h**) Representative images and quantification of NG2^+^ cell viabilities using PI exclusion assay in OPC enriched cultures after ± light treatment at 108 kJ/m^2^ light dose. All above histograms are normalized to controls with data representing means ± s.e.m. of a number of biological replicates (n indicated on each histogram) and *p* values from Student’s two tailed unequal variance t-test. Black and blue histograms represent conditions kept in the dark or exposed to light respectively, and dashed lines are control values. Media conditions for experiments are described above their respective images. Light doses in kJ/m^2^ are shown above histograms and as insets within representative images of cells treated with light.

To address the effects of light on non-transfected cells (see methods), we developed a customized plate housing 6 light emitting diodes (LEDs) emitting blue light (470 nm) that sits on top of a standard 6 well culture dish with a controllable output power (*W*: 0.1–1.5 mW/mm^2^ at cell surface), flash duration (τ: 1–10 ms) and frequency (*f*: 0.1–90 Hz), operated from a power unit housed outside the incubator (Fig. 1a). Optogenetics is intensively utilized in neuroscience, so we began by irradiating primary rat cortical neurons with flashes of light at intensities typically used to activate ChR2 transfected neurons (*W* = 1 mW/mm^2^, τ = 5 ms, and *f* = 1 Hz) for 20 hours^21, 22^. This generates a light dose we express here as Joules per square meter, thus the aforementioned stimulation protocol equates to 360 kJ/m^2^ (see methods), a similar light dose to that used for optogenetic gene regulation^17, 18^, optogenetic directed stem cell induction^23^ and live-cell imaging studies^24, 25^. At this dose of light, we find that mature rat cortical neurons, cultured for 21 days *in vitro* (d.i.v.) at high density, display a significant loss of neuronal viability after exposure to light compared to control cells kept in the dark (p = 0.02; Fig. 1b), detected by propidium iodide (PI) exclusion assay (see methods). However, immature neurons, 7 d.i.v., were significantly more sensitive to light (p = 0.00004; Fig. 1b). We excluded heat as a possible cause of cell death by regulating the temperature of the incubator to maintain 37 °C in the media of the plates during light stimulations using thermocouple measurements, indicating that the effects are directly due to light exposure.

We next examined the effects of light on other CNS cell types. Using mixed glial cultures which contain a dense astrocyte sublayer with oligodendrocyte progenitor cells (OPCs) and microglia on top, we observed very few cells permeable to propidium iodide (PI) in all cultures, however variations in glial fibrillary acidic protein (GFAP) staining for astrocytes frequently occurred in cultures treated with light (Fig. 1c). Closer examination of NG2^+^ OPCs identified a decrease in OPC viability in cultures exposed to light (p = 0.03; Fig. 1c). To examine changes in astrocytes after light treatment more closely, we returned to our 7 d.i.v. cortical neuron enriched cultures, which contain 5.75 ± 1.9% GFAP^+^ astrocytes, for better cellular resolution. There were no significant changes in the numbers of astrocytes between conditions, but the morphology of astrocytes treated with light was less ramified and with the appearance of GFAP blebbing along processes (Fig. 1d). To quantify this, we used ImageJ plugins NeuronJ and Sholl Analysis to manually trace and quantify astrocyte intersection numbers respectively, at increasing radii from their nuclei (Fig. 1e). This revealed robust and significant (p < 0.0001) changes in astrocyte morphologies by comparing the mean intersection numbers of astrocytes with or without light (Fig. 1f).

Recently, blue light has been shown to alter mouse microglial cells *in vitro* ^26^. Microglia are the major phagocytic cells in the CNS; we separated and enriched them from mixed glial cultures by agitation and preferential adhesion, yielding a population of cells that were 87.3 ± 7.5% positive for Isolectin-B4 (IB4). We exposed microglia to high doses of light (792 kJ/m^2^) by irradiating the cells for 44 hours (*W* = 1 mW/mm^2^, τ = 5 ms, and *f* = 1 Hz). Microglia viability was unaffected, but we detected a significant increase (p = 0.017) in microglia cell volume when treated with light (Fig. 1g). This indicates that microglia have a higher tolerance to blue light and enter a potentially altered state of activation when irradiated, in agreement with previous observations^26^.

To confirm that the loss of OPCs from mixed glia after light treatment (Fig. 1c) was not due to changes in the other cells present in mixed glial cultures, we enriched OPCs (86 ± 1.8% positive for NG2) using agitation followed by microglial depletion. Exposing OPCs to lower levels of light power of 0.3 mW/mm^2^ but keeping the same pulse frequency and light duration delivers ~a third (108 kJ/m^2^) of the light dose used in the mixed glia and neuronal (360 kJ/m^2^) experiments. This caused a highly significant (p = 0.0002) decrease in OPC viability (Fig. 1h). Together, these data show that all CNS cells are altered by light *in vitro*, and that OPCs are particularly sensitive. OPCs are highly proliferative, migratory cells that generate all the myelinating oligodendrocytes in the CNS^27^. Due to their broad range of cellular properties i.e. proliferation, migration and differentiation, combined with their particular sensitivity to light, we used the OPC as a model cell to investigate light toxicity.

### Solving *in vitro* light toxicity

**Figure 2:**
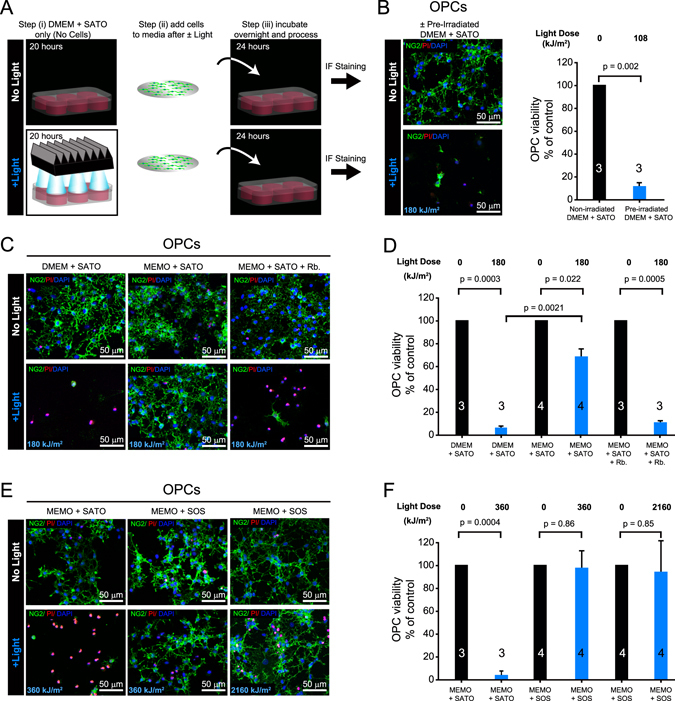
*In vitro* photo-toxicity can be resolved with a new culture medium. (**a**) Setup of experiment to test media for photo-reactive components. Step (i) media is treated ± light stimulation (*W* = 0.3 mW/mm^2^, t = 5 ms and *f* = 1 Hz) for 20 hours delivering 108 kJ/m^2^ of light, step (ii) after which viable cells are placed in ± pre-irradiated media for 24 hours before step (iii) analysis by PI exclusion assay. (**b**) Viabilities of OPCs 24 hours after placement into DMEM + SATO treated ± light (108 kJ/m^2^). Note: the robust loss of OPC viability in pre-irradiated media is similar to irradiating cells directly with light (see Fig. 1h). (**c** and **d**) Significant improvement (p = 0.0021) of OPC viability in photo-inert media MEMO + SATO compared to DMEM + SATO when treated with light (180 kJ/m^2^), but both conditions retain significant loss of viabilities compared to cells kept in dark (p = 0.0003 and p = 0.022 respectively), and addition of riboflavin (Rb.), a component absent in MEMO, restores highly significant loss (p = 0.0005) of OPC viability when treating cells with light. (**e** and **f**) Increasing light dose to 360 kJ/m^2^ induces robust loss of OPC viability in MEMO + SATO, however, combining MEMO + SOS protects OPC viabilities during light doses of 360 kJ/m^2^ and up to 2160 kJ/m^2^ (*W* = 0.6 mW/mm^2^, t = 0.5 ms and *f* = 10 Hz), a dose 20 fold greater than the 108 kJ/m^2^ dose that induced almost complete loss of OPC viability in standard conditions of DMEM + SATO (see Fig. 1h). All above histograms are normalized to controls with data representing means ± s.e.m. of a number of biological replicates (value within or above each histogram) and *p* values from Student’s two tailed unequal variance t-test. Black and blue histograms represent conditions kept in the dark or exposed to light respectively. Light doses in kJ/m^2^ are shown above histograms and as insets within representative images of irradiated experiments. Cell culture media conditions are described above images and under respective histograms.

Light can cause changes to both media and to the intracellular components within cells. To assess whether there are toxic factors in the media generated by light, we placed viable OPCs into media (DMEM + SATO) that was previously irradiated with blue light (108 kJ/m^2^) (Fig. 2a). Examining OPC viability after 24 hours in pre-irradiated media yielded the same degree of photo-toxicity to that which was observed by light stimulating the cells directly (Fig. 2b), thus demonstrating that the culture media is the principal source of photo-damage. We reformulated DMEM for our experiments by removing photo-reactive components and created Modified Eagle’s Medium for Optogenetics (MEMO). Replacing DMEM with MEMO in OPC cultures during irradiation with a light dose of 180 kJ/m^2^ improved cell viability from 5 ± 1.6% to 69 ± 7.1% after irradiation (Fig. 2c and d). Riboflavin (vitamin B2) is one of the principal components in DMEM responsible for the photo-damaging effects, as reintroducing it into MEMO (Fig. 2c and d) closely resembled the level of cell death detected in DMEM conditions after treatment with light. Increasing the light intensities to amounts that induced loss of neuronal viabilities (360 kJ/m^2^) recapitulated the loss of OPC viability in MEMO based media (Fig. 2e and f). This indicates the need for further photo-protective supplements in addition to the removal of photo-reactive components from the media. The photo-inert MEMO opened up the possibility of screening for such cell culture media additives that would specifically rescue light induced cell death.

The cellular sensitivity to photo-toxicity, from above results, correlates with the cellular expression of glutathione (GSH) (where microglia express over 8 times more GSH than astrocytes^28^, and astrocytes express 2–3 times more GSH than cortical neurons^29–31^ and OPCs^32^); that and because the amount of reactive oxygen species (ROS) generated by light are dependent upon riboflavin concentrations^33^, argues for the possibility of making media supplements that would protect cells against cellular photo-toxicity. Thus, we formulated Supplements for Optogenetic Survival (SOS), to complement our photo-inert media. SOS was designed based upon available information on serum free supplements of NS21^34^, N2^35^ and B27^36^ formulations with slight modifications, additional vitamins, and antioxidants, as well as the removal of thyroxine (T4) and triiodo-l-thyronine (T3), because T4 and especially T3 are potent agonists of OPC differentiation^37^. Combining MEMO with SOS during irradiation prevented all signs of photo-damage in OPCs treated with 360 kJ/m^2^ of light (p = 0.86; Fig. 2e and f). To determine the limit of our photo-protective media we exposed OPCs to high dosages of light (*W* = 0.6 mW/mm^2^, t = 0.5 ms and *f* = 10 Hz) for 20 hours to simulate high frequency optogenetic stimulation. There was no loss of viability observed (p = 0.85; Fig. 2e and f) despite delivering a light dose 20 times higher (2160 kJ/m^2^) than the one which previously caused a complete loss of OPC viability in standard conditions (Fig. 1h).

**Figure 3:**
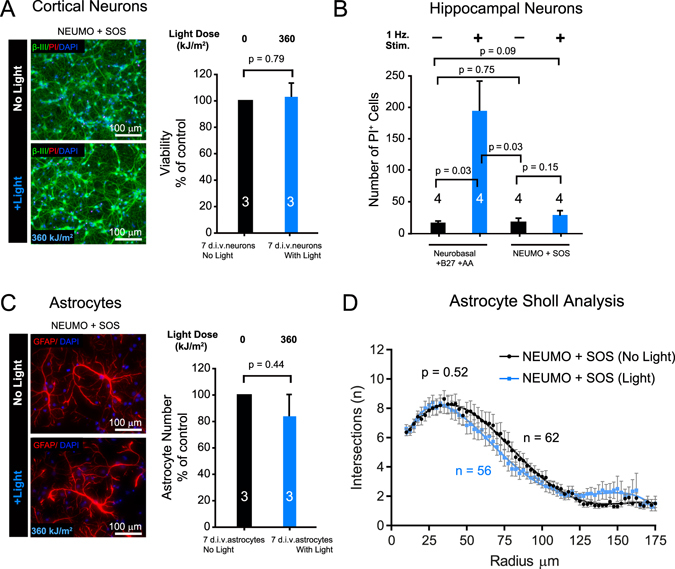
Light induced cytotoxicity can be prevented with new culture medium. (**a**) Viability of 7 d.i.v. β-III^+^ cortical neurons ± light at indicated dose in NEUMO (in replacement of Neurobasal) and SOS (in replacement of B27) using PI exclusion assay. (**b**) Number of PI positive cells in 7 d.i.v. hippocampal neuronal cultures ± optogenetic stimulation at 1 Hz in control media conditions (Neurobasal + B27) with additional antioxidants (AA) and in NEUMO + SOS. Note: the protection of cortical and hippocampal neurons during treatment with light only in NEUMO + SOS media conditions. (**c**) Representative images and quantification of GFAP^+^ astrocyte numbers in 7 d.i.v. cortical neuron enriched cultures after ± light at indicated dose in NEUMO + SOS. (**d**) Non-linear fitted plots from Sholl Analysis of astrocytes kept in the dark (black line: 62 cells analyzed) or exposed to light (blue line: 56 cells analyzed) and *p* value calculated from two tailed unpaired t-test of mean intersection number. All above histograms are normalized to controls (with the exception of **b**) and data represents means ± s.e.m. of a number of biological replicates (value within or above each histogram) and *p*-values from Student’s two tailed unequal variance t-test. Black and blue histograms represent conditions kept in the dark or exposed to light respectively. Light doses in kJ/m^2^ are shown above histograms and as insets within representative images of irradiated experiments. Cell culture media conditions are described above images and under respective histograms.

Following on from these results, we formulated NEUronal Media for Optogenetics (NEUMO) to be used in combination with SOS for neuronal cells. Using the more light sensitive 7 d.i.v. cultures of enriched cortical neurons and exposing them to 360 kJ/m^2^ light, we observed complete protection of neuronal viability (Fig. 3a). Using 7 d.i.v. hippocampal neurons (Fig. 3b) and simulating optogenetic stimulations at 1 Hz and intensities of 1 mW/mm^2^ used for *in vitro* optogenetic experiments, showed increased neuronal cell death in standard conditions of Neurobasal medium with B27 and supplemented with additional antioxidants (AA), which previously have been shown to prevent significant cell death in mature hippocampal neurons when light stimulated^22^. However, NEUMO and SOS media showed no increased neuronal cell death under these conditions (Fig. 3b). Furthermore, no change in astrocyte numbers (p = 0.44) or morphology (p = 0.52) was detected in 7 d.i.v. cortical cultures treated with or without light in NEUMO with SOS (Fig. 3c and d).

### New media confers photo-protection

**Figure 4:**
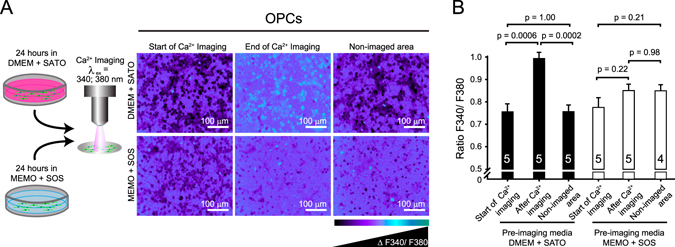
Preventing photo-toxicity during calcium imaging. (**a**) Schematic of OPCs taken from DMEM + SATO based media and MEMO + SOS based media for ratiometric Fura2-AM Ca^2+^-imaging (λ_ex_ = 340; 380 nm: UV light) and the representative images acquired at the start, at the end of Ca^2+^ imaging and in non-imaged areas at the end of experiment. (**b**) Ratiometric Ca^2+^ changes in OPCs at the start, after imaging or in non-imaged areas after OPCs were pre-treated in DMEM + SATO (closed histograms) or MEMO + SOS (open histograms) based media. Histograms show means ± s.e.m. of a number of biological replicates (value within or above each histogram) and *p*-values from Student’s two tailed unequal variance t-test.

Other common techniques that expose cells to potentially damaging light dosages are live cell imaging and ion sensitive imaging, particularly calcium imaging, as Fura2-AM based Ca^2+^ imaging uses high energy UV light excitation at wavelengths of 340 and 380 nm. Calcium ions are major signal transduction modulators in all cells and their temporal and spatial regulation is of great importance to the neuroscience field^38^. Using OPCs, we observed significant calcium increases in imaged regions when compared to the start of the experiment (p = 0.0006) or to non-imaged areas (p = 0.0002) (Fig. 4a and b), despite the imaging being performed in photo-inert Ringer’s buffer. This may be due to cell damage from UV exposure over the course of the 84 ± 2.5 minutes of imaging at ~1 frame per second on average, with an exposure time of 200 ms. We hypothesized that maintenance of cells in standard media formulations may sensitize them to photo-toxicity even after cells are removed from their maintenance media, which was DMEM + SATO in our case. In contrast, we reasoned that pre-incubation of cells with the antioxidant rich SOS might confer protection against light damage. To test this hypothesis, we placed OPCs into MEMO + SOS for ~24 hours prior to Ca^2+^ imaging. We then imaged the cultures for an average of 83.8 ± 2 minutes in Ringer’s buffer at an average of ~1 frame per second and measured calcium levels at the start and end of the experiment as well as in non-imaged regions. Using MEMO and SOS we prevented significant changes in calcium levels in imaged areas compared to the start (p = 0.22) or to in non-imaged areas (p = 0.98) (Fig. 4a and b), supporting the idea that pre-incubation of cells in MEMO + SOS can prove beneficial, by limiting the effects of photo-sensitization and conferring a degree of photo-protection. Taken together, our results show that by modifying conventional culture medium and supplements, photo-toxicity can be nearly eliminated.

**Figure 5:**
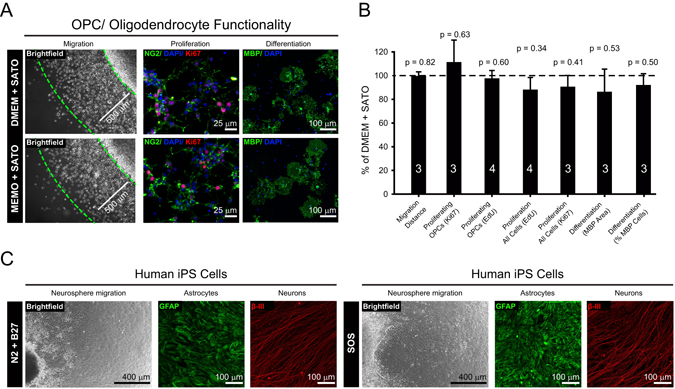
The new media preserves normal cell function. (**a**) Comparison of the effect of DMEM + SATO and MEMO + SATO based media upon OPC migration over 4 days from agarose drop (Brightfield images), OPC proliferation using NG2 and Ki67 staining after 3 days with mitogens PDGF-aa and FGF-b (middle panel) and OPC differentiation with terminal differentiation marker MBP after 5 days of mitogen withdrawal and treatment with T3 (right panel). (**b**) Data from (**a**) analyzed by average distances from edge of drop to corona of migrated cells, percentages of NG2^+^ cells with Ki67^+^ or EdU^+^ and percentages of MBP^+^ cells and percentage MBP area generated for differentiation, and all normalized to DMEM + SATO values with data representing means ± s.e.m. of a number of biological replicates (value within or above each histogram) and *p*-values from Student’s two tailed unequal variance t-test. (**c**) Comparison of neural epithelial cell differentiation from human iPSC generated embryonic bodies using serum free supplements N2+B27 (left hand panels) or SOS (right hand panels) to generate neurons (β-III) and astrocytes (GFAP) after approximately 5 weeks *in vitro*.

Riboflavin is an essential co-factor for numerous physiological reactions. Thus, we tested if its absence from MEMO media would impact OPC migration, proliferation and differentiation over time. To study migration, we used an agarose drop assay^39^, whereby high densities of OPCs are suspended in low melting point agarose and seeded as 1.5 µl drops onto glass coverslips. Over a period of four days OPCs migrate radially outward from the edge of the drop to form a corona of migrating OPCs. Measuring the average distance of the corona from the edge of the drop showed no difference in MEMO + SATO compared to DMEM + SATO media (Fig. 5a and b). For proliferation analysis we examined the percentages of NG2^+^ OPCs that expressed nuclear localized Ki67, a marker of cellular proliferation (interphase), and also separately for EdU incorporation into the DNA, a marker of S-phase (or DNA replication), after 3 days in the presence of mitogens. We detected no difference in MEMO or DMEM based media (Fig. 5a and b). Removal of mitogens and addition of the thyroid hormone T3 promotes robust OPC terminal differentiation into mature MBP^+^ oligodendrocytes. We detected no difference between MEMO and DMEM media over 5 days of differentiation by examining the percentage of cells expressing MBP or the size of differentiated cells using MBP area (Fig. 5a and b). These observations are in line with previous findings that complete B vitamin removal from media for a week had no effect on HEK-293T cell morphology, attachment and proliferation^40^. Although riboflavin is essential for numerous cellular metabolic activities, the 1 µM concentration of riboflavin in DMEM and Neurobasal media may be saturating for primary OPCs and loads them with sufficient levels to perform normal cellular functions for days in the absence of exogenous riboflavin.

The SOS formulation is based upon B27^36^, NS21^34^ and N2^35^ components. Embryonic and induced pluripotent stem cells (iPSCs) are frequently maintained in chemically defined conditions supplemented with N2 + B27 for many weeks, so we sought to determine if our SOS formulation was comparable to N2 + B27 by analyzing neuronal and astrocytic genesis from terminally plated human iPSC derived embryonic bodies^41^. After five weeks of differentiation we observed a similar size, shape and number of neurospheres and similar densities of β -III^+^ neurons and GFAP^+^ astrocytes generated using either SOS or N2 + B27 supplementation (Fig. 5c). This establishes that our SOS formulation is a viable alternative for long term serum free cell culturing, thus facilitating the use of live cell imaging and optogenetics on iPSC models of human disease. Taken together, our new media can be universally used for experiments dependent on light and our modified supplements can completely replace conventional media supplements.

## Discussion

We show here an economical, efficient and easily accessible solution to photo-toxicity with broad applications to neuroscience and beyond. We demonstrate the benefit of the media for optogenetics, live-cell and ratiometric Ca^2+^ imaging, by focusing on neural cells. However, light induced toxicity has been well documented for a number of other cell types: (HeLa^9^, HK^42^, RPE^43, 44^, U2OS^9^, U373-MG^25^, CHO-K1^24^, V79^1^, NIH-3T3^42^, 3T6-DF8^1^, and COS-7^9^). Thus, these media compositions have the potential to provide photo-protection on diverse types of cells, even to high doses of light over 2000 kJ/m^2^ on cells as sensitive as OPCs. Our solution to photo-toxicity will also be pertinent to popular super-resolution live cell microscopy methods that require light intensities ranging from 10 mW/mm^2^ to 1x10^4^ mW/mm^2^ at the sample^45^. Moreover, recent findings showed the beneficial use of NEUMO and SOS media for fluorescence-activated cell sorting (FACS)^46^.

We developed the media by screening DMEM and Neurobasal formulations^36^ for photo-reactive components to generate our own photo-inert media, MEMO and NEUMO, as replacement media for experiments using light. Such screening approaches have been successfully applied to generate customized media for GFP photo-stability^40^ and neuronal electrophysiology^47^ *in vitro*. Blue light has been shown to alter cell viability through flavin containing oxidases^42^, damage to mitochondrial DNA^48^ and lipofuscins^43, 44^ within cells. The knowledge gained from these experiments was applied to the available formulations of NS21 and N2 to generate SOS, a viable alternative to other serum free supplements which, when combined with MEMO or NEUMO, shifts the photon budget currently imposed by standard cell culture conditions. Whether our study has implications to *in vivo* experiments, is uncertain, as blood flow and brain interstitial fluid exchange may be rapid enough to clear toxic by-products and prevent build up, thus preventing the phototoxic effects observed *in vitro*. Nevertheless, as *in vitro*, the effects of visible light on tissue will be limited to the intensity, wavelength and duration of light exposure^9, 24, 25, 49, 50^. Although the main focus on the potential side-effects of light stimulations *in vivo* has been on thermal changes, photo-chemical by-products from visible light excitation of lipofuscins^43, 44^ flavoproteins^42^, porphyrin containing proteins^51^, and cellular pigments such as melanin and neuromelanin^52^ could have detrimental effects. To what level it is possible to reduce photo–toxicity and ROS generation *in vivo*, as we have shown here for *in vitro* is unclear. However, our study highlights the importance of controlling for potential photo-toxicity, ROS generation and other off target effects of light stimulation; such as proliferation, cell death or morphological changes of cells in light exposed areas for both *in vivo* and *in vitro* light stimulation experiments.

Current methods for limiting photo-toxicity *in vitro*, as well as *in vivo*, are mainly centered on hardware setup such as controlling delivery of light^8, 53^, using less energetic wavelengths of light^7, 9^ or changing the experimental approach. The media and supplements described here provide an economical way to prevent or limit the undesirable effects of light *in vitro*, to improve experimental quality, and maximize the potential of available optical tools and those in development in an easily accessible manner.

## Materials and Methods

### Media formulation

Modified Eagle’s Medium for Optogenetics (MEMO) and NEUronal Media for Optogenetics (NEUMO) were developed by screening available formulations for DMEM and Neurobasal for photo-reactive components using cell viability assays and literature searches. Supplements for Optogenetic Survival (SOS) is formulated and based upon the components of the serum free supplements B27^36^, NS21^34^ and N2^35^ with removal of triiodo-L-thyronine (T3) and thyroxine (T4) and with additional proprietary modifications. MEMO, NEUMO and SOS were made in house, or sourced from Cell Guidance Systems; UK, as part of their LiveLight package and used according to manufacturer’s instructions.

### Cell-culture

Unless otherwise stated all compounds were sourced from Sigma-Aldrich; UK. All cells were cultured at 37 °C in a humidified atmosphere containing 5% CO_2_. Primary mixed glial cultures and OPCs were prepared as described previously^54^. Briefly, brains from P2 postnatal rats were dissected, meninges removed, cortices chopped and digested with papain (Worthington Biochemical Corporation, USA) for 1 hour, followed with ovomucoid (0.5 mg/ml BSA, 1 mg/ml trypsin inhibitor, 40 µg/ml DNase I Type IV, 1% Penicillin and Streptomycin (Pen/Strep) in DMEM), trituration, and seeded as 3 cortical hemispheres per poly-D-lysine (PDL) coated T-75 flasks or glass coverslips. Mixed glia were grown for 10–12 days *in vitro* (d.i.v.) in DMEM with 10% fetal calf serum (FCS) (Life Technologies; UK) and 1% Pen/Strep. OPCs and microglia were separated from astrocytes by mechanical dissociation (175 r.p.m. on ~19 mm diameter (Ø) orbital shaker) for 16 hours at 37 °C. Microglia were enriched using adherence to non-treated dishes (Corning; UK) (15 ml/10 cm plate) for 15 min, and OPCs enriched by centrifuging (250*g*_*max*_ for 8 minutes) the remaining suspension. OPCs were resuspended in DMEM with modified SATO (100 µg/ml BSA, 60 ng/ml progesterone, 16.1 µg/ml putrescine, 5 ng/ml sodium selenite, 5 µg/ml insulin, 5 µg/ml N-acetyl-L-cysteine, 50 µg/ml holo-transferrin and 1% Pen/Strep) and growth factors (PDGF-aa at 10 ng/ml and FGF-b at 10 ng/ml; Peprotech; UK). OPCs were seeded at a density of 22 × 10^3^ cells/cm^2^ onto PDL coated glass coverslips or flasks with daily addition of growth factors yielding 86 ± 1.8% (n = 18) NG2^+^ cells after 3 days of proliferation. Microglia were dissociated from non-treated dishes with TrypLE Express (Life Technologies; UK), and seeded onto 22 mm Ø PDL coated glass coverslips (450,000 cells/coverslip) in 10% FCS, 1% Pen/Strep in DMEM yielding 87.3 ± 7.5% (n = 5) IB4^+^ cells. For primary neurons, cortices from E18 rat embryos were chopped and digested with papain for 15 min followed with ovomucoid in DMEM or Ca^2+^ and Mg^2+^ free EBSS, and then triturated using fire polished glass pipettes. Cells were clarified over a 4% BSA step in ovomucoid and seeded onto PDL coated glass coverslips in 6 well plates at 1 × 10^6^ cells per plate in neuronal medium (Neurobasal, 2% B27 and 1% Glutamax; Life Technologies; UK) with 1% Pen/Strep and supplemented with 5% FCS. The next day media was changed to neuronal medium without FCS yielding 80 ± 2.8% β-III-tubulin^+^ neurons and 5.75 ± 1.9% GFAP^+^ astrocytes (n = 3). None of the cells we examined were modified to express light sensitive proteins such as ChR2, or recombinant proteins (such as GFP).

### OPC proliferation, migration, and differentiation assays

OPCs were plated onto PDL coated coverslips in DMEM + SATO with growth factors (PDGF-aa and FGF-b) left overnight and placed into either DMEM + SATO or MEMO + SATO, with daily mitogen addition for 3 days. To measure proliferation, fixed cells were immunostained for NG2 and Ki67 co-localization. Alternatively, 100 nM EdU was applied 24 hours prior to fixation and detected according to manufacturer’s instructions (Life Technologies; UK). Migration assays were performed using an agarose drop assay^39^. OPCs were proliferated, dissociated and re-seeded as 1.5 µl drops of 1 × 10^6^ cells/µl in 1% w/v low melting point agarose, onto PDL and matrigel (BD Biosciences; UK) coated coverslips. Plated drops were cooled at 4° C for 15 min, then 50 µl of 10% FCS in DMEM added, and the well flooded with MEMO + SATO or DMEM + SATO media, with growth factors, and fixed 4 days later. Migration distance (distance from the drop edge to the corona of migrated cells) was calculated from five photo-micrographs of each drop taken with a Zeiss AxioVision digital microscope. Data shown are of at least three replicates. For differentiation experiments, OPCs on PDL coated coverslips in DMEM + SATO media with T3 (400 ng/ml), were placed in either DMEM + SATO + T3 or MEMO + SATO + T3 and cells allowed to differentiate for 5 days. Terminal differentiation was detected with MBP immunostaining as described below.

### iPSC differentiation

Human induced pluripotent stem cells (hiPSCs) following proliferation with FGF-2 (20 µg/ml) and heparin (2 µg/ml), were treated with a combination of retinoic acid (100 nM) and the sonic-hedgehog analog, puromorphamine (PM, 1 µM) for 15 days. Cells were then cultured as an embryonic body (EB) suspension in PM for another 11 days. After completion of this stage, cells were kept in DMEM:F12 composition media containing glia stimulating growth factors (60 ng/ml T3; 10 ng/ml PDGF-aa; 10 ng/ml IGF1; 10 ng/ml NT3; 1 µM cAMP; and 100 ng/ml d-biotin) for 50–100 d.i.v. Terminal plating of gliogenic EBs was on poly-ornithine/laminin-coated glass coverslips. Terminally plated iPSC-derived gliogenic EBs were grown in a DMEM:F12 formulation media with either N2 + B27 or SOS for five weeks, then fixed and stained for GFAP and β-III-tubulin as below.

### Light toxicity assays

Cells were irradiated with blue light (470 ± 10 nm) emitted from an LED plate; controlling power (*W*: 0.1–1.5 mW/mm^2^ at cell surface), flash duration (τ: 1–10 ms) and frequency (*f*: 0.1–90 Hz) from an electronic stimulator housed outside the incubator (Fig. 1a). Light power was measured using a LaserCheck photo-diode (Coherent; UK). Incubator temperature was adjusted to maintain 37 °C in the cell culture media using thermocouple measurements and placing the 6 well plate onto a metal heat dissipater. For light toxicity media comparison experiments, cells were transferred into DMEM or MEMO based media with mitogens for OPCs, or Neurobasal or NEUMO based media for neurons and astrocytes, for 4 hours prior to light treatment and with either SATO, 2% B27 or SOS. For the 2160 kJ/m^2^ OPC stimulation experiments, cells were plated on PDL and matrigel coated coverslips and allowed to proliferate for three days, before transferring coverslips into MEMO + SOS media with growth factors, and allowed to equilibrate for 4 hours before light treatment. Non-viable cells were detected by adding 10 µg/ml propidium iodide (PI) (Life-Technologies; UK) to cells for 20 min prior to fixation.

### Immunocytochemistry and fluorescence microscopy

All washes and incubation steps were performed in PBS with 0.01% w/v sodium azide (PBS-NaN_3_) unless otherwise stated. Cell cultures were fixed for 15 min at RT in 4% w/v paraformaldehyde (PFA) in PBS. Fixed cells were blocked and permeabilized with 0.1% Triton X-100 with 10% v/v goat serum (Vector Laboratories; UK) in PBS for 1 hour at RT. Primary and secondary antibody sources and dilutions are shown in Table 1. Primary antibody incubations were overnight at 4° C, followed with PBS wash, secondary antibody incubations at RT for 1 hour followed with PBS wash, stained for 10 min at RT with DAPI or Hoechst-33342 (Life Technologies; UK) followed with PBS wash prior to mounting in Mowiol with 2.5% w/v DABCO. Omission of primary antibodies was used to verify specificity as control in all experiments. Photo-micrographs were taken with using Leica SP5 confocal or Leica DM inverted microscopes (Leica; UK).

**Table 1:** .

Primary Antibodies			
**Target**	**Source**	**Species**	**Dilution**
NG2	Millipore; UK	Rabbit	1:200
NG2	Millipore; UK	Mouse	1:100
MBP	Serotec; UK	Rat	1:100
IB4-Alexa-488*	Life-Technologies; UK	N/A	1:100
Ki67	Thermo-Scientific; UK	Rabbit	1:200
β -III-Tubulin	Sigma-Aldrich; UK	Mouse	1:100
GFAP	Dako: UK	Rabbit	1:500
GFAP	Sigma-Aldrich; UK	Mouse	1:500
**Secondary Antibodies**			
**Target**	**Source**	**Fluorophore-**λ_**ex**_	**Dilution**
Goat-Anti-Rabbit	Life-Technologies; UK	Alexa-488	1:1000
Goat-Anti-Mouse	Life-Technologies; UK	Alexa-488	1:1000
Goat-Anti-Rat	Life-Technologies; UK	Alexa-488	1:1000
Goat-Anti-Rabbit	Life-Technologies; UK	Alexa-555	1:1000
Goat-Anti-Mouse	Life-Technologies; UK	Alexa-555	1:1000
Goat-Anti-Rabbit	Life-Technologies; UK	Alexa-647	1:500
Goat-Anti-Mouse	Life-Technologies; UK	Alexa-647	1:500

*Isolecithin-B4 (IB4) is a lectin conjugated to the Alexa-488 fluorophore, N/A; not applicable, λ_ex_; excitation wavelength in nm.

### Image analysis

Binarised images of MBP and IB4 were used to calculate fraction areas with ImageJ v1.48, and normalized to controls for each biological replicate. For Sholl analysis, GFAP images were binarised and branches manually traced using the NeuronJ plugin in ImageJ followed with Sholl Analysis v3.4.1 plugin in ImageJ^55^. Sholl parameters were 10 µm starting radius with 2.5 µm steps. Viability was calculated as cell numbers with DAPI and cell specific marker (NG2 or β-III-tubulin) minus those with PI, and values normalized to control conditions.

### Calcium Imaging

Coverslips with OPCs were placed in either DMEM + SATO or MEMO + SOS with mitogens for ~24 hrs, prior to loading with 4 µM Fura2-AM (Life Technologies; UK) for 1 hour at 37 °C. Coverslips were placed on an Olympus IX71 microscope superfused with buffered Ringer’s solution containing the following (in mM) 124 NaCl, 2.5 KCl, 2 MgCl_2_, 1 NaH_2_PO_4_, 26 NaHCO_3_, 10 Glucose and 2.5 CaCl_2_ and bubbled with 95% O_2_, 5% CO_2_. Fluorescent images from 340 nm and 380 nm excitations were collected, on average from all experiments (n = 10), for 83.9 ± 1.54 minutes at an average of ~1 frame per second (0.78 ± 0.02), with an exposure time of 200 ms. The emission of Fura2-AM is measured at 510 nm after excitation at 340 nm and 380 nm and the ratio of these emission intensities correlates with the calcium concentration within the cell.

### Statistical Analysis

Numbers of experiments are indicated on bargraphs, data shown as mean ± standard error of the mean (s.e.m.), and assumed to follow normal distribution. P values from Student’s two tailed unequal variance t-tests < 0.05 were considered significant.
